# The cation channel Trpa1 and chemokine Cxcl1 mediate axonal degeneration in spared nerve injury–induced neuropathic pain

**DOI:** 10.1016/j.jbc.2025.110654

**Published:** 2025-08-30

**Authors:** Liu-Qing Ye, Xuan-Xuan Huang, Hua-Feng Liu, Tian Li, Yu Wang, Xiao-Hui Chen, Yu Zheng, Zuo-Ming Chen, Qin-Yu Liu, Fan Yang, Nan-Qi Li, Li Wan

**Affiliations:** 1 Department of Pain Medicine, The State Key Specialty in Pain Medicine, The Second Affiliated Hospital, Guangzhou Medical University, Guangzhou, P.R. China; 2 Stem Cell Translational Medicine Center, The Second Affiliated Hospital, Guangzhou Medical University, Guangzhou, P. R. China; 3 Department of Anesthesiology, Zhujiang Hospital of Southern Medical University, Guangzhou, P.R. China; 4 Department of Anesthesiology, Sun Yat-sen University Cancer Center, State Key Laboratory of Oncology in Southern China, Collaborative Innovation for Cancer Medicine, Guangzhou, P.R. China

**Keywords:** neuropathic pain, Schwann cell, macrophage, *Trpa1*, *Cxcl1*

## Abstract

Demyelination of peripheral nerve injury is a vital cause of neuropathic pain. Schwann cells play an important role in supporting and maintaining the removal and regeneration of myelin debris from neuronal axons in the peripheral nervous system. Creating a good immune microenvironment would promote the Schwann cells to repair the injured nerve and reverse the allodynia of neuropathic pain. *Trpa1*, a transient receptor potential ion channel, and *Cxcl1*, a chemokine, play crucial roles in allodynia in pain pathophysiology. However, their interaction in neuropathic pain remains unclear. This study aimed to elucidate the role of *Trpa1* in neuropathic pain and its interaction with *Cxcl1*. Using a spared nerve injury model in mice, mechanical allodynia was observed alongside increased *Trpa1* expression in injured nerves. Treatment with a *Trpa1* inhibitor alleviated allodynia, suggesting *Trpa1* is involved in neuropathic pain. Transcriptome sequencing revealed immune pathway enrichment post-*Trpa1* inhibition. *Cxcl1* recruited *Cxcr2*-positive macrophages to injured sites, whereas *Trpa1* inhibition reduced *Cxcl1* expression and *Cxcr2* recruitment in Schwann cells and in neurons. In addition, *Mag* (myelin-associated glycoprotein), crucial for axonal stability, was downregulated in the spared nerve injury model but increased post-*Trpa1* blockade, indicating *Trpa1* plays an important role in the *Cxcl1*-mediated immune cascade in axonal degeneration. In summary, neuronal and dedifferentiated Schwann cell *Trpa1* regulates *Cxcl1* synthesis, recruiting macrophages to nerve injury sites and mediating neuropathic pain. Collectively, our findings suggest that *Trpa1* activation weakens axonal resistance to degeneration by inhibiting *Mag*. This study highlights the multifaceted involvement of *Trpa1* in neuropathic pain, suggesting a potential therapeutic target for neuropathic pain management.

Peripheral nerve injury is frequently encountered in clinical medicine, involving damage or abnormalities to peripheral nerves, which may be caused by trauma, infection, or other reasons. Poor repair or reconstruction of nerves can lead to neuropathic pain, which is characterized by spontaneous pain, hyperalgesia, allodynia, and sensory abnormalities (1). Persistent pain not only disrupts patients' quality of life but also seriously influences the patients’ work, sleep, and social activities, increasing the incidence of emotional disorders, such as depression and anxiety. The current treatment for neuropathic pain is inadequate, as about half of the patients do not experience sufficient pain relief. This may be due to a lack of understanding of the mechanisms involved (2), particularly the crosstalk between glia and neurons in nerve repairing. Therefore, exploring the pathogenesis of neuropathic pain is the key to promoting the recovery of damaged nerves and alleviating the pain of patients with neuropathic pain.

Schwann cells, a type of glial cell in peripheral nerves, play a crucial role in maintaining and regenerating neurons. Their main functions include providing nourishment and insulation to individual nerve fibers (3). Research indicates that Schwann cells play a significant role in maintaining pain hypersensitivity (4). For example, following peripheral nerve injury, Schwann cells exhibit a series of pathophysiological reactions to provide an appropriate environment for nerve regeneration (5).

*Trpa1*, a transient receptor potential ion channel, is expressed in sensory neurons, glial cells, and epithelial cells. It plays a significant role in the pathophysiological processes of pain and inflammation (6). Studies have reported that Schwann cells can express *Trpa1* and maintain pain hypersensitivity and neuroinflammation through the reactive oxygen species oxidative stress pathway (7). However, whether activation of *Trpa1* in Schwann cells is related to the secretion of chemokines or cytokines has not been reported. Chemokines play crucial roles in inflammation, immune responses, and the neural environment following neuronal damage (8). Chemokines also participate in neuroimmune modulation, influencing the interaction between neurons and immune cells (9). This involvement may contribute to the persistence and exacerbation of neuropathic pain. Combining the aforementioned information, we hypothesize that upon peripheral nerve injury, activation of *Trpa1* on Schwann cells promotes the secretion and release of chemokines, recruiting immune cells to the site of injury to participate in maintaining pain hypersensitivity. Whether Schwann cells can modulate the chemokine expression by *Trpa1* is unclear following nerve injury.

Neuropathic pain is related to abnormal expression or activation of ion channels, including activation of the *Trpa1* channel (10). In addition, neurons can release various transmitters and factors, including *Ngf* (nerve growth factor), *Bdnf* (brain-derived neurotrophic factor), *Ccl2* (chemokine [C–C motif] ligand 2), and others (11). These transmitters and chemokines may directly participate in the regulation of inflammation and neuropathic pain (12). Therefore, we hypothesize that upon peripheral nerve injury, activation of *Trpa1* on neurons promotes the secretion and release of chemokines, recruiting immune cells to the site of injury to participate in maintaining pain hypersensitivity. Whether the neuron-expressing *Trpa1* mediated the chemokine expression remains unclear. First of all, we hypothesize that upon peripheral nerve injury, activation of the *Trpa1* channel on neurons and Schwann cells promotes the secretion and release of chemokines, recruiting immune cells to the site of injury to create an inflammatory microenvironment to participate in maintaining pain hypersensitivity. In this study, we established a spared nerve injury (SNI) neuropathic pain model using C57BL/6J mice. Through experiments such as transcriptome sequencing, RT–quantitative PCR (RT–qPCR), tissue section immunofluorescence staining, and protein immunoblotting, we aim to elucidate the relationship between *Trpa1* on neurons and chemokines on Schwann cells, as well as their impact on axonal degeneration and pain hypersensitivity.

## Results

### Increased *Trpa1* activity accounts for the increased pain sensitivity following sciatic nerve injury

**Figure 1:**
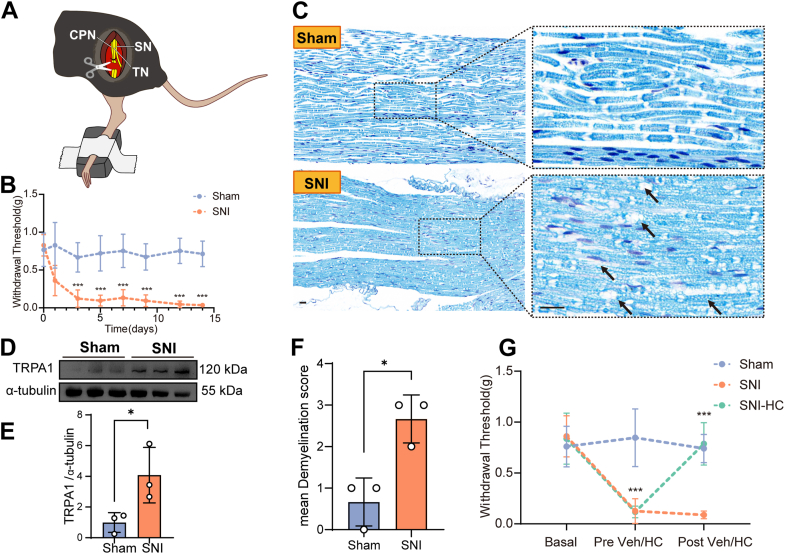
**Activation of *Trpa1* in the sciatic nerve induces mechanical allodynia in SNI mice.***A*, schematic representation of the SNI model in mice. *B*, mechanical paw withdrawal threshold measurements in mice. n = 10, ∗∗∗*p* < 0.001, two-way ANOVA followed by Bonferroni post hoc analyses. *C*, Luxol Fast Blue (LFB) staining of the sciatic nerve in mice. The scale bar represents 100 μm. *D*, protein expression of *Trpa1* in the sciatic nerve of mice (n = 3 per group). *E*, statistical analysis of *Trpa1* protein expression in the sciatic nerve of mice, *p* = 0.0496 < 0.05, unpaired two-tailed Student’s *t* test. *F* attributed to the mean demyelination score assessed by LFB staining of sections from the sciatic nerve, n = 3 per group, *p* = 0.0132 < 0.05, unpaired two-tailed Student’s *t* test. *G*, mechanical paw withdrawal threshold measurements in mice. n = 10, ∗*p* < 0.05, two-way ANOVA followed by Bonferroni post hoc analyses. CPN, common peroneal nerve; SN, sural nerve; SNI, spared nerve injury; TN, tibial nerve.

To investigate the mechanism of *Trpa1* in neuropathic pain, we established a mouse model of neuropathic pain induced by SNI (Fig. 1*A*). Compared with the Sham group, the von Frey mechanical pain threshold was significantly decreased on day 3 and persisted to day 14 following SNI treatment (Fig. 1*B*), indicating the establishment of mechanical hypersensitivity in injured mice. Luxol Fast Blue staining was performed to assess the integrity and necrosis degree of myelin under neuropathic pain. We observed irregular, swollen, twisted, fractured, and vacuolated myelin, indicating demyelination of the sciatic nerve and organic pathological changes in the injured nerve (Fig. 1, *C* and *F*). Next, we determined the protein expression of *Trpa1* in the sciatic nerve. Western blotting revealed that SNI induced a significant increase in *Trpa1* protein expression in the sciatic nerve (Fig. 1, *D* and *E*). Furthermore, to confirm the role of *Trpa1* in neuropathic pain, we treated mice with sciatic nerve injury (SNI-HC group) with the *Trpa1* inhibitor HC-030031 (100 mg/kg, i.p.). The results showed that HC-030031 significantly alleviated the mechanical allodynia induced by SNI (Fig. 1*G*). These results demonstrated that peripheral nerve injury increased the expression of *Trpa1* channels in damaged nerves, leading to the development of mechanical hypersensitivity.

### The increases of *Trpa1* regulated the immune pathways in neuropathic pain

**Figure 2:**
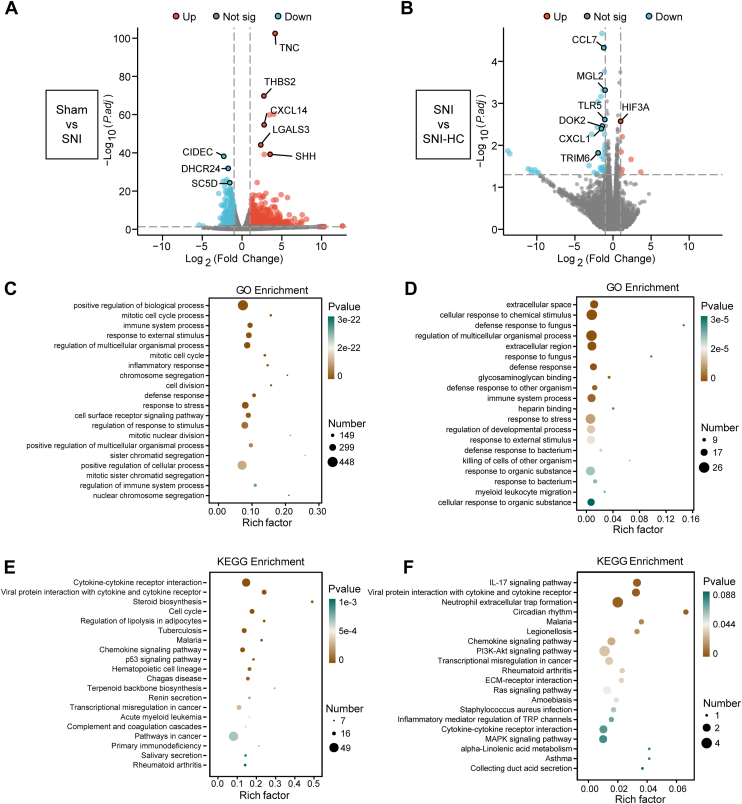
**Differential gene expression analysis and enrichment pathways in the injured sciatic nerve.***A*, volcano plot showing a comparison of the distribution of differentially expressed genes (DEGs) between Sham and SNI groups. *Red dots* represent upregulated genes, *blue dots* represent downregulated genes, and *gray dots* represent genes with no significant difference in expression. *B*, volcano plot showing the comparison of the distribution of DEGs between SNI and SNI-HC groups. *Red dots* represent upregulated genes, *blue dots* represent downregulated genes, and *gray dots* represent genes with no significant difference in expression. *C* and *D*, bubble plot showing the top 20 significantly enriched pathways obtained from Gene Ontology (GO) enrichment analysis of DEGs between the Sham and SNI groups (*C*) and between the SNI and SNI-HC groups (*D*). *E* and *F*, bubble plot showing the top 20 significantly enriched pathways obtained from Kyoto Encyclopedia of Genes and Genomes (KEGG) enrichment analysis of DEGs between the Sham and SNI groups (*E*) and between the SNI and SNI-HC groups (*F*). Pathways with more enriched DEGs are represented by larger bubbles in *C*–*F*. The color of the *bubbles* indicates the significance level based on the *p* value. SNI, spared nerve injury.

To explore the potential mechanisms of *Trpa1*-mediated neuropathic pain, we performed RNA-Seq analysis on the injured-side sciatic nerves of the Sham, SNI, and SNI-HC groups. The differential gene expression was investigated before and after *Trpa1* inhibition. Statistical analysis of significant differentially expressed genes (DEGs) was performed. The volcano plot illustrated alterations in gene expression, with upregulated genes such as *Tnc* (tenascin), *Thbs2* (thrombospondin-2), *Lgals3* (lectin, galactoside-binding, soluble, 3), *Shh* (sonic hedgehog protein), and *Cxcl14* (chemokine [C–X–C motif] ligand 14), whereas downregulated genes included *Cidec* (cell death inducing DFFA like effector c), *Dhcr24* (delta[24]-sterol reductase), and *Sc5d* (sterol-C5-desaturase) in the Sham *versus* SNI group (Fig. 2*A*). In the SNI *versus* SNI-HC group, upregulated genes included *Hif3a* (hypoxia-inducible factor 3-alpha) and *Pmp2* (Myelin P2 protein), whereas downregulated genes included *Ccl7* (chemokine [C–C motif] ligand 7), *Mgl2* (macrophage galactose-type C-type lectin 2), *Cxcl1* (chemokine [C–X–C motif] ligand 1), and *Tlr5* (toll-like receptor 5) (Fig. 2*B*). To determine the major biological functions of DEGs, we conducted Gene Ontology (GO) enrichment analysis. In the Sham *versus* SNI comparison, the top 20 enriched biological processes were mostly associated with inflammation and immune responses (Fig. 2*C*). In the SNI *versus* SNI-HC comparison, the top enriched biological processes included cellular responses to chemical stimuli, regulation of multicellular organismal processes, and immune system processes (Fig. 2*D*). Downregulated genes accounted for 90% of the total DEGs. These results indicate that mice in the SNI group initiated inflammatory defense responses to injury stimuli, whereas these responses were variably inhibited after treatment with the *Trpa1* inhibitor. This suggests that *Trpa1* plays a role in promoting immune activity and enhancing inflammatory responses, and these responses are among the mechanisms underlying neuropathic pain. We also performed Kyoto Encyclopedia of Genes and Genomes (KEGG) pathway enrichment analysis using clusterProfiler to determine the enrichment of DEGs in metabolic pathways. In the Sham *versus* SNI comparison, enriched metabolic pathways included cytokine–cytokine receptor interactions and the P53 signaling pathway (Fig. 2*E*). In the SNI *versus* SNI-HC comparison, enriched metabolic pathways included the IL-17 signaling pathway and chemokine signaling pathway. These findings further support the role of *Trpa1* in mediating neuropathic pain through immune pathways (Fig. 2*F*).

### *Trpa1* is involved in immune pathways through Schwann cells, neurons, and macrophages

**Figure 3:**
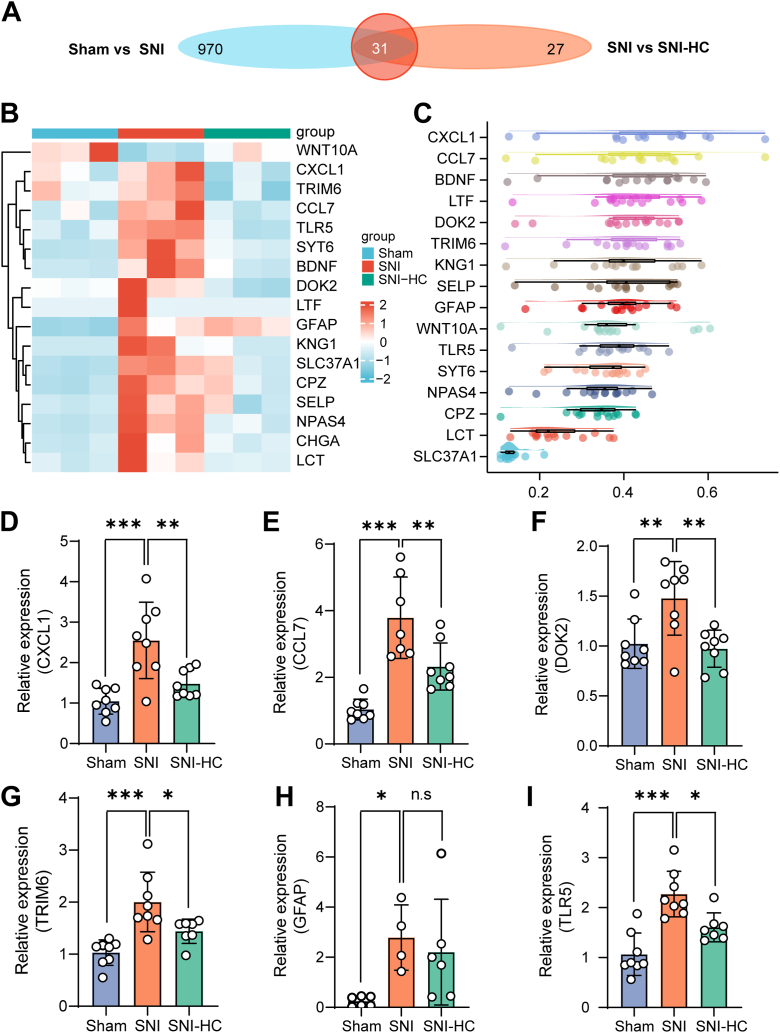
**Relationship between differentially expressed genes (DEGs) and macrophages, neurons, Schwann cells.***A*, Venn diagram showing the number of shared and unique DEGs between the Sham *versus* SNI and SNI *versus* SNI-HC comparison groups. *B*, the heat map is showing the expression pattern of the top overlapping DEGs between the Sham *versus* SNI group and SNI *versus* SNI-HC group, with *blue* signifying downregulated genes and *red* signifying upregulated genes. *C*, gene similarity analysis shows the degree of similarity of top differentially overlapping expressed genes. *D*–*I*, the mRNA expression levels of *Cxcl1* (one-way ANOVA, *F* = 12.96, Tukey's multiple comparisons test, ∗∗∗*p* < 0.001, ∗∗*p* < 0.01) (*D*), *Ccl7* (one-way ANOVA, *F* = 21.53, Tukey's multiple comparisons test, ∗∗∗*p* < 0.001, ∗∗*p* < 0.01) (*E*), *Dok2* (one-way ANOVA, *F* = 8.044, Tukey's multiple comparisons test, ∗∗*p* < 0.01) (*F*), *Trim6* (one-way ANOVA, *F* = 12.54, Tukey's multiple comparisons test, ∗∗∗*p* < 0.0001, ∗*p* < 0.05) (*G*), *Gfap* (one-way ANOVA, *F* = 4.466, Tukey's multiple comparisons test, ∗*p* < 0.05) (*H*), and *Tlr5* (one-way ANOVA, *F* = 18.07, Tukey's multiple comparisons test, ∗∗∗*p* < 0.001, ∗*p* < 0.05). (*I*) in mouse sciatic nerve tissue (Sham group: n = 8, SNI group: n = 6–8, SNI-HC group: n = 6–8). *ns* represents no statistical difference. SNI, spared nerve injury.

To investigate the specific pathways in which *Trpa1* participates in immune responses, we employed Venn diagrams to illustrate the number of common and unique DEGs among the comparison groups. This approach effectively illustrates both the count of DEGs and the overlap between the comparison groups. Notably, we identified a total of 31 DEGs present in both the Sham *versus* SNI and SNI *versus* SNI-HC comparison groups (Fig. 3*A*). The heat map shows the expression changes of differentially overlapping expressed genes (Fig. 3*B*). Among these genes, 90% exhibited upregulation after nerve injury and downregulation upon *Trpa1* inhibition. Gene similarity analysis shows the degree of similarity of top differentially overlapping expressed genes. The results suggested *Cxcl1* can be potential key regulatory genes for *Trpa1* regulation in neuropathic pain (Fig. 3*C*). Furthermore, genes such as *Tlr5*, *Ccl7*, *Trim6* (tripartite motif-containing protein 6), *Cxcl1*, *Dok2* (docking protein 2), and *Gfap* (glial fibrillary acidic protein) were identified as being associated with neurons, macrophages, or Schwann cells. These genes are either produced by neuron, Schwann cells, or macrophages, and chemoattract macrophages to inflammatory sites. RT–qPCR results confirmed the upregulation of these genes after nerve injury and downregulation upon *Trpa1* inhibition (Fig. 3, *D*–*I*). Therefore, our results indicate that *Trpa1* activates immune pathways mainly through neurons, Schwann cells, or macrophages in the peripheral nervous system following nerve injury.

### *Trpa1* is expressed in dedifferentiated Schwann cells

**Figure 4:**
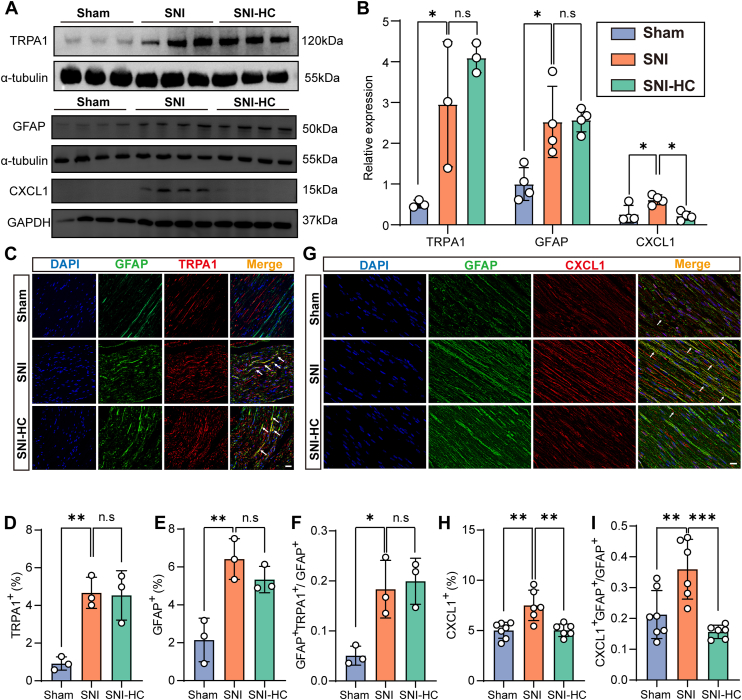
**Immune pathways of *Trpa1* in dedifferentiated Schwann cells.***A*, protein expression levels in mouse sciatic nerve tissue: Protein expression levels of *Trpa1* in mouse sciatic nerve tissue (Sham group: n = 3, SNI group: n = 3, and SNI-HC group: n = 3). Protein expression levels of *Gfap* in mouse sciatic nerve tissue (Sham group: n = 4, SNI group: n = 4, and SNI-HC group: n = 4). Protein expression levels of *Cxcl1* in mouse sciatic nerve tissue (Sham group: n = 4, SNI group: n = 4, and SNI-HC group: n = 4). *B*, statistical analysis of *Trpa1* (one-way ANOVA, *F* = 12.07, Tukey's multiple comparisons test, ∗*p* < 0.05), *Gfap* (one-way ANOVA, *F* = 9.497, Tukey's multiple comparisons test, ∗*p* < 0.05) and *Cxcl1* (one-way ANOVA, *F* = 7.895, Tukey's multiple comparisons test, ∗*p* < 0.05) protein expression levels in mouse sciatic nerve tissue. *C*, immunofluorescence staining of mouse sciatic nerve tissue: DAPI (*blue*), *Gfap* (*green*), and *Trpa1* (*red*). The scale bar represents 20 μm. *D*, statistical analysis of *Trpa1*-positive expression in mouse sciatic nerve tissue (n = 3 per group, one-way ANOVA, *F* = 16.17, Tukey's multiple comparisons test, ∗∗*p* < 0.01). *E*, statistical analysis of *Trpa1Gfap*-positive expression in mouse sciatic nerve tissue (n = 3 per group, one-way ANOVA, *F* = 14.98, Tukey's multiple comparisons test, ∗∗*p* < 0.01). *F*, statistical analysis of the proportion of double-positive expression of *Trpa1* and *Gfap* to total *Gfap*-positive expression in mouse sciatic nerve tissue (n = 3 per group, one-way ANOVA, *F* = 10.36, Tukey's multiple comparisons test, ∗*p* < 0.05). *G*, immunofluorescence staining of mouse sciatic nerve tissue: DAPI (*blue*), *Gfap* (*green*), and *Cxcl1* (*red*). The scale bar represents 20 μm. *H*, statistical analysis of *Cxcl1*-positive expression in mouse sciatic nerve tissue (n = 6–7 per group, one-way ANOVA, *F* = 11.68, Tukey's multiple comparisons test, ∗∗*p* < 0.01). *I*, statistical analysis of the proportion of double-positive expression of *Cxcl1* and *Gfap* to total *Gfap*-positive expression in mouse sciatic nerve tissue (n = 6–7 per group, one-way ANOVA, *F* = 12.39, Tukey's multiple comparisons test, ∗∗*p* < 0.01, ∗∗∗*p* < 0.001). *ns* represents no statistical difference. DAPI, 4′,6-diamidino-2-phenylindole; SNI, spared nerve injury.

Studies have shown that HC-030031 inhibits *Trpa1* channel function without affecting the expression of channel proteins. Consistent with our experimental results (Fig. 4, *A* and *B*), the protein expression level of *Trpa1* remained unchanged after inhibitor treatment. Under pathological conditions of nerve injury, Schwann cells can dedifferentiate from mature myelinating cells to a dedifferentiated state, facilitating nerve repair and regeneration. The present study showed that mRNA levels of *Gfap*, as a marker for dedifferentiated Schwann cells, significantly increased in the SNI group, whereas there was no significant change in mRNA levels of *Gfap* in the SNI-HC group (Fig. 3*H*). Further validation at the protein expression level revealed a consistent trend with mRNA expression (Fig. 4, *A* and *B*), indicating that Schwann cells dedifferentiated in response to nerve injury stimulation, but the use of HC-030031 has no significant effect on the expression of dedifferentiated Schwann cells. To investigate whether *Trpa1* mediates immune responses through dedifferentiated Schwann cells, we found that dedifferentiated Schwann cells express *Trpa1* (Fig. 4*C*). In addition, fluorescence ratio analysis revealed a concurrent upregulation of *Trpa1* expression with increased expression in dedifferentiated Schwann cells (Fig. 4, *D* and *E*). Colocalization statistics showed that dedifferentiated Schwann cells in the SNI group expressed more *Trpa1* than the Sham group (Fig. 4*F*). These results indicate that proliferating dedifferentiated Schwann cells can express *Trpa1*, mediating immune pathways in response to nerve injury.

### Dedifferentiated Schwann cells regulate the secretion and release of *Cxcl1* through *Trpa1*

To investigate the specific mechanism by which *Trpa1* mediates the immune pathway through dedifferentiated Schwann cells, we analyzed the RNA-Seq results. Compared with the Sham group, the expression of the chemokine *Cxcl1* was upregulated in the SNI group. Importantly, HC-030031 treatment significantly inhibited the upregulation of chemokine *Cxcl1* induced by SNI. We confirmed the consistency of *Cxcl1* changes at both mRNA and protein levels through RT–qPCR and Western blot experiments (Figs. 3*D* and 4, *A* and *B*). *Cxcl1* is a chemokine that attracts inflammatory cells to inflammatory sites, and its chemotactic effect regulates the inflammatory environment (12). To investigate whether *Cxcl1* is involved in the *Trpa1* immune pathway, the immunofluorescence staining showed that *Cxcl1* were expressed in the dedifferentiated Schwann cells, and the *Cxcl1* level in the dedifferentiated Schwann cells was significantly increased following SNI (Fig. 4, *G*–*I*). These results indicate that the increased expression of *Trpa1* in dedifferentiated Schwann cells promotes the synthesis and release of their own *Cxcl1*, thereby mediating the immune pathway after nerve injury.

### Neurons regulate the secretion and release of *Cxcl1* through *Trpa1*

**Figure 5:**
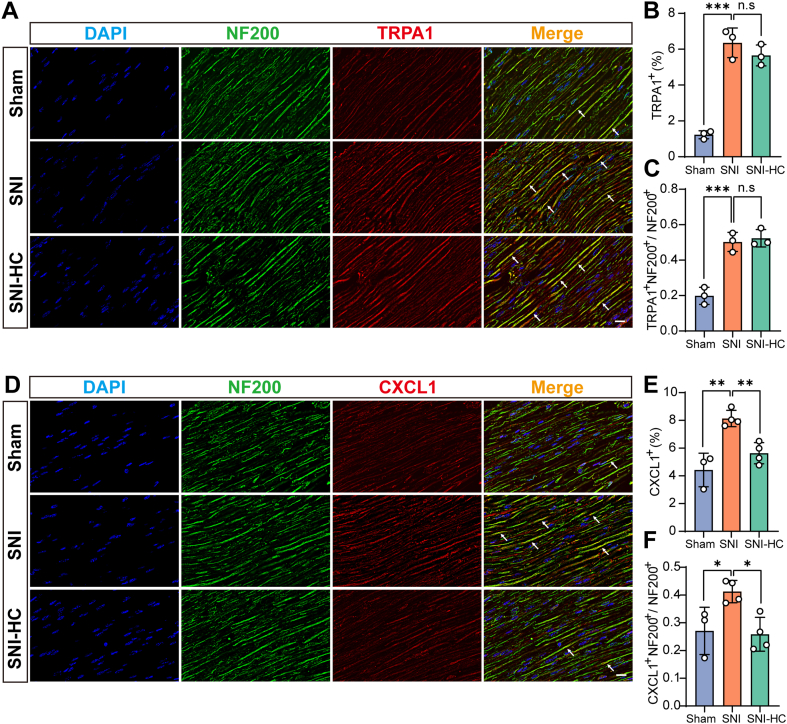
**Immune pathways of *Trpa1* in injured nerve neurons.***A*, immunofluorescence staining of mouse sciatic nerve tissue: DAPI (*blue*), NF200 (*green*), and *Trpa1* (*red*). The scale bar represents 20 μm. *B*, statistical analysis of *Trpa1*-positive expression in mouse sciatic nerve tissue (n = 3 per group, one-way ANOVA, *F* = 64.41, Tukey's multiple comparisons test, ∗∗∗*p* < 0.001). *C*, statistical analysis of the proportion of double-positive expression of *Trpa1* and NF200 to total NF200-positive expression in mouse sciatic nerve tissue (n = 3 per group, one-way ANOVA, *F* = 38.71, Tukey's multiple comparisons test, ∗∗∗*p* < 0.001). *D*, immunofluorescence staining of mouse sciatic nerve tissue: DAPI (*blue*), NF200 (*green*), and *Cxcl1* (*red)*. The scale bar represents 20 μm. *E*, statistical analysis of *Cxcl1*-positive expression in mouse sciatic nerve tissue (n = 3–4 per group, one-way ANOVA, *F* = 17.96, Tukey's multiple comparisons test, ∗∗*p* < 0.01). *F*, statistical analysis of the proportion of double-positive expression of *Cxcl1* and NF200 to total NF200-positive expression in mouse sciatic nerve tissue (n = 3–4 per group, one-way ANOVA, *F* = 7.407, Tukey's multiple comparisons test, ∗*p* < 0.05). *ns* represents no statistical difference. DAPI, 4′,6-diamidino-2-phenylindole.

In the peripheral nervous system, besides Schwann cells, neurons also express *Trpa1*, and studies have shown that neurons can release a range of factors (11). To investigate the involvement of neuronal *Trpa1* in the immune pathway, the immunofluorescence costaining of the neuronal marker NF200 (Neurofilaments) and *Trpa1* (Fig. 5*A*) was first done. The *Trpa1* immunofluorescence proportion was consistent with previous studies (Fig. 5*B*), then the neurons in the SNI group expressed more *Trpa1* than that of in the Sham group (Fig. 5*C*). Next, we detected whether the neuronal *Trpa1* is involved in the *Cxcl1* expression. The results showed that the expression of chemokine *Cxcl1* is colocalized with the NF-200-positive nerve fibers (Fig. 5*D*). The *Cxcl1* immunofluorescence proportion was also consistent with previous studies (Fig. 5*E*). Following the HC-030031 application, the expression of *Cxcl1* in NF-200-positive fiber was significantly reduced compared with the SNI group (Fig. 5*F*). These results indicate that the neuronal *Trpa1* also promotes the synthesis and release of *Cxcl1* in neurons following SNI.

### *Cxcl1* attracts *Cxcr2*^+^ macrophages to the injured nerves

**Figure 6:**
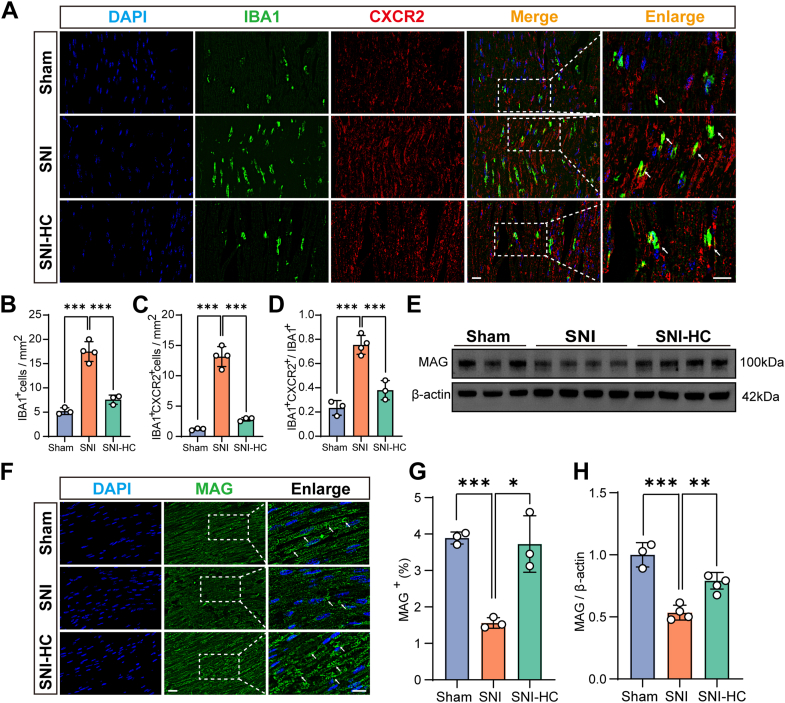
***Trpa1* increases infiltration of *Cxcr2*+ macrophages and expression of *Mag* in injured nerves.***A*, immunofluorescence staining of mouse sciatic nerve tissue: DAPI *(blue*), *Iba1* (*green*), and *Cxcr2* (*red)*. The scale bar represents 20 μm. *B* and *C*, statistical analysis of *Iba1*-positive (n = 3–4 per group, one-way ANOVA, *F* = 69.39, Tukey's multiple comparisons test, ∗∗∗*p* < 0.001) (*B*) and double-positive expression of *Iba1* and *Cxcr2* (n = 3–4 per group, one-way ANOVA, *F* = 127.0, Tukey's multiple comparisons test, ∗∗∗*p* < 0.001) (*C*) cell expression in mouse sciatic nerve tissue. *D*, statistical analysis of the proportion of double-positive expression of *Iba1* and *Cxcr2* to total *Iba1*-positive cell expression in mouse sciatic nerve tissue (n = 3–4 per group, one-way ANOVA, *F* = 46.78, Tukey's multiple comparisons test, ∗∗∗*p* < 0.001). *E*, protein expression levels of *Mag* in mouse sciatic nerve tissue (Sham group: n = 3, SNI group: n = 4, and SNI-HC group: n = 4). *F*, immunofluorescence staining of mouse sciatic nerve tissue: DAPI (*blue*) and *Mag* (*green*). The scale bar represents 20 μm. *G*, statistical analysis of *Mag*-positive expression in mouse sciatic nerve tissue (n = 3 per group, one-way ANOVA, *F* = 34.71, Tukey's multiple comparisons test, ∗∗∗*p* < 0.001, ∗*p* < 0.05). *H*, statistical analysis of *Mag* protein expression levels in mouse sciatic nerve tissue (n = 3–4 per group, one-way ANOVA, *F* = 34.76, Tukey's multiple comparisons test, ∗∗∗*p* < 0.001, ∗∗*p* < 0.01). DAPI, 4′,6-diamidino-2-phenylindole; SNI, spared nerve injury.

The aforementioned results indicate that nerve injury upregulates the *Cxcl1* expression in both Schwann cells and neurons simultaneously. In addition, we found differential expression of macrophage-related genes after the RNA-Seq analysis. The macrophage was significantly increased in the SNI group (Fig. 6, *A* and *B*). Moreover, following HC-030031 treatment, the macrophage upregulation induced by SNI was reversed (Fig. 6, *A* and *B*), indicating that after nerve injury, macrophages are recruited to the damaged site by inflammatory factors. To further investigate the relationship between *Trpa1* and macrophages, the *Cxcl1* receptor *Cxcr2* (C–X–C chemokine receptor type 2) was costained with macrophages. The results showed that the *Cxcr2*-positive signal was expressed in the macrophages (Fig. 6*A*) compared with the SNI group. Application of HC-030031 significantly inhibited the *Cxcr2*^+^ macrophage upregulation induced by SNI (Fig. 6, *C* and *D*). Our results indicate that *Trpa1* promotes the synthesis and release of *Cxcl1*, attracting peripheral *Cxcr2*^+^ macrophages to the injury site to mediate the immune pathway after nerve injury.

### *Trpa1* reduces axonal stability and resistance to degeneration

*Mag* (myelin-associated glycoprotein) is a glycoprotein in the myelin sheath. It is primarily expressed on the myelin sheath in the nervous system, which is produced by Schwann cells, and is responsible for wrapping and maintaining the axons of neurons. *Mag* helps maintain the stability of microtubules in axons in the peripheral nervous system and serves as a marker of axonal resistance to degeneration. After SNI, we observed a significant decrease in *Mag* expression but a significant increase in *Mag* expression after HC treatment (Fig. 6, *E* and *H*). Immunofluorescence experiments showed the consistent results like the protein detection (Fig. 6, *F* and *G*). These results indicate that nerve injury leads to a decrease in *Mag* synthesis and expression, resulting in reduced resistance of axons to damage and degeneration. Blocking *Trpa1* contributes to the synthesis and expression of *Mag*, which, combined with its stabilizing axons and resisting degeneration, suggests that *Trpa1* impairs axonal stability and resistance to degeneration.

## Discussion

In this study, through an SNI-induced neuropathic pain model, we demonstrated that the *Trpa1* activation in neurons and dedifferentiated Schwann cells plays an important role in allodynia after nerve injury. Activation of *Trpa1* in Schwann cells and neurons regulates the synthesis and release of *Cxcl1*, recruiting *Cxcr2*-positive macrophages to the site of nerve injury to further aggravate the immune microenvironment, resulting in neuropathic pain. *Trpa1* suppresses *Mag*, weakening axonal microtubule stability and reducing resistance to degeneration. Our finding indicates that a *Trpa1*–*Cxcl1* axis mediates axonal degeneration in Schwann cells and neurons in spare nerve injury–induced neuropathic pain and offers a new therapeutic strategy and theoretical insights for treating neuropathic pain.

Neuropathic pain is a chronic condition often associated with nerve damage, neural inflammation, or neurological disorders (13). Studies by Liu *et al.* (14) have found that following sciatic nerve injury in mice, there is disruption, disintegration, and demyelination of the injured nerve myelin, potentially leading to nerve fiber exposure. This loss of myelin may contribute to the development of mechanical allodynia and hyperalgesia in the mouse hind paw (15); these findings align with the results of our study.

*Trpa1* is a transient receptor potential ion channel that can be activated by a wide range of noxious external stimuli, participating in the mechanisms of pain and inflammation (16, 17). Martínez-Rojas *et al.* (18) found that *Trpa1* expression in the dorsal root ganglion was upregulated in a model of pain induced by complete Freund’s adjuvant injection into the paw. Cui *et al.* (19) also observed increased *Trpa1* expression in the trigeminal ganglion of mice in a partial infraorbital nerve injury pain model (pT-ION), and blocking *Trpa1* reversed tactile allodynia. In this study, we employed the SNI model to simulate neuropathic pain. We found that *Trpa1* expression increased in the injured sciatic nerve, and inhibition of *Trpa1* improved tactile allodynia behavior in mice.

Transcriptome sequencing facilitates a comprehensive investigation into gene function and structure at the whole-genome level, enabling the identification of DEGs within cells, tissues, or individuals across various physiological or pathological conditions. Descalzi *et al.* (20) conducted transcriptome sequencing analysis of the dorsal root ganglion, medial prefrontal cortex, and anterior cingulate cortex in the SNI model to identify specific and meaningful genetic targets for the diagnosis and treatment of neuropathic pain (20, 21, 22, 23). However, transcriptome sequencing of sciatic nerve tissue has not been reported. In our study, we conducted transcriptome sequencing analysis of the sciatic nerves from Sham, SNI, and SNI-HC groups. Results showed significant changes in gene expression in injured nerves postinjury, involving various physiological processes, such as inflammation, stress, and lipid metabolism (24, 25). Nerve injury results in mechanical disruption of cells and tissues, leading to the release of numerous inflammatory mediators and lipid substances. This may affect the structure of myelin sheaths and lipid metabolism. Furthermore, in the GO and KEGG analyses, our analysis showed alterations in immune inflammation and nutrient metabolism pathways postnerve injury, suggesting their involvement in peripheral neuropathic pain. In mice with nerve injury treated with a *Trpa1* inhibitor, we found 31 shared DEGs, potentially key in *Trpa1*-mediated neuropathic pain. GO enrichment analysis revealed enrichment in defense response, immune system processes, stress responses, and responses to external stimuli. KEGG pathway analysis revealed enrichment in pathways such as the IL-17 signaling pathway, chemokine signaling pathway, regulation of inflammatory mediators on transient receptor potential channels, PI3K–Akt signaling pathway, cytokine–cytokine receptor interaction, and mitogen-activated protein kinase signaling pathway. These signaling pathways are all linked to immune-inflammatory processes, indicating that *Trpa1* may mediate neuropathic pain *via* immune pathways. Further analysis of the 31 shared DEGs, such as *Tlr5*, *Ccl7*, *Trim6*, *Cxcl1*, *Dok2*, and *Gfap*, revealed associations with neurons, macrophages, or Schwann cells (26, 27, 28, 29, 30, 31, 32, 33, 34, 35), indicating their potential involvement in the *Trpa1*-mediated immune pathway. Our gene similarity analysis shows the degree of similarity of top differentially overlapping expressed genes, and the results suggested *Cxcl1* can be potential key regulatory genes for *Trpa1* regulation in neuropathic pain. *Trpa1* expression in neurons and Schwann cells could modulate the immune pathway by regulating chemokines in the peripheral nervous system.

Schwann cells in the peripheral nervous system support and protect nerve fibers, promoting nerve regeneration. Injuries can disrupt their barrier function, leading to abnormal nerve impulse conduction. In response to injury, Schwann cells switch from myelinating to demyelinating states. They aid in clearing damaged nerve fragments and promoting axon regeneration (36). De Logu *et al.* (37) found that the role of Schwann cells in neuropathic pain may be immunologically related. In our study, we observed increased *Trpa1* expression in dedifferentiated Schwann cells following nerve injury. These cells also secrete *Cxcl1*, with levels decreasing significantly upon *Trpa1* inhibition. This suggests that *Trpa1* in dedifferentiated Schwann cells mediates neuropathic pain by regulating *Cxcl1* secretion after nerve injury. After nerve injury, neurons express growth-related molecules like brain-derived neurotrophic factor to support axonal plasticity and survival (38). In addition, they release factors crucial for neural development, repair, and neuropathology (39). Notably, in neuropathic pain models, neurons release *Cxcl1*, linked with pain transmission (40). *Trpa1* is found in many tissues, including the nervous system, where it is primarily in sensory neurons involved in sensing mechanical and chemical stimuli (41). Our study demonstrated increased *Trpa1* and *Cxcl1* expression on nerve fibers postinjury. *Trpa1* inhibition reduced *Cxcl1* expression, implying the role of *Trpa1* in regulating *Cxcl1* synthesis and release by neurons, potentially contributing to neuropathic pain.

Therefore, we can conclude that peripheral nerve injury upregulates *Trpa1* channels in neurons and dedifferentiated Schwann cells, leading to *Cxcl1* release. This *Cxcl1* increase triggers inflammation around neurons, fostering neuropathic pain development.

*Cxcl1* plays a crucial role in inflammation and tissue injury (42). It acts as a chemoattractant for inflammatory cells like neutrophils and macrophages, directing their migration to the inflamed site (43, 44, 45). Studies by Ntogwa *et al.* (34) have found that peripheral injection of *Cxcl1* can induce pain-like behavior in mice. Mai *et al.* (46) found that after peripheral nerve injury, a large number of immunocytes infiltrate the site of nerve injury. In our experiment, macrophage numbers significantly increased at the injured nerve site of SNI model mice after nerve injury. The neurons and dedifferentiated Schwann cells produce and release substantial amounts of *Cxcl1*. Moreover, we observed a significant increase in *Cxcr2*-positive macrophages, indicating recruitment mediated by *Cxcl1*. Treatment with a *Trpa1* inhibitor led to a notable reduction in both total infiltrating macrophages and *Cxcr2*-positive macrophages. This indicates that the release of *Cxcl1* guides the recruitment of *Cxcr2*-positive macrophages. *Cxcl1* attracts macrophages to the injured nerve site, worsening neuropathic pain by releasing inflammatory mediators that affect neuronal excitability (47, 48, 49, 50). This cycle sustains neuropathic pain presence.

Our findings delineate a pathway wherein *TRPA1* activation induces *Cxcl1* synthesis to promote infiltration of *Cxcr2*-positive macrophages, ultimately driving mechanical allodynia in neuropathic pain. However, this study has defined boundaries in mechanistic resolution. Although we established *Cxcl1* as the critical chemokine mediating macrophage recruitment, direct validation of *Cxcl1*–*Cxcr2* axis functionality remains to be completed, for example, through administration of recombinant *Cxcl1* with concurrent *Cxcr2* antagonist blockade. Furthermore, the relative contribution of *Cxcr2*^*+*^ macrophages *versus* other immune effectors—particularly microglia expressing *Cxcr2* cognate receptors—warrants clarification *via* cell-specific depletion approaches, whereas temporal dynamics of this signaling cascade require finer resolution beyond the current single-timepoint sampling after nerve injury. Crucially, these parameters do not compromise our primary conclusion regarding *Trpa1*–*Cxcl1* signaling but instead focus future investigations on key extensions.

**Figure 7:**
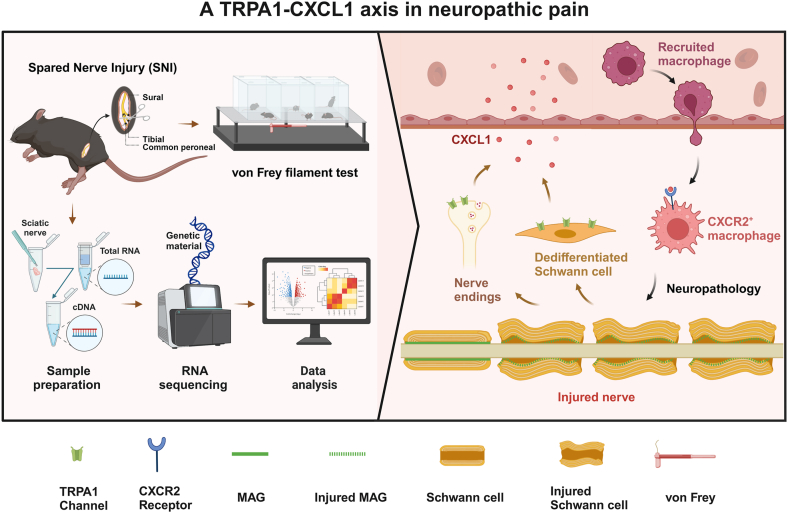
**Mechanism diagram of the *Trpa1*–*Cxcl1* axis in neuropathic pain.** Neurons and dedifferentiated Schwann cells mediate the synthesis and release of *Cxcl1* through *Trpa1*, recruiting *Cxcr2*-positive macrophages to the site of nerve injury to further aggravate the immune microenvironment, resulting in neuropathic pain. *Trpa1* suppresses *Mag*, weakening axonal microtubule stability and reducing resistance to degeneration.

*Mag*, produced by Schwann cells, is a key component of myelin in the peripheral nervous system. It supports axonal growth, neuronal migration, and interactions between axons and myelin, thus maintaining myelin stability and impacting neuron structure and function (51, 52, 53). Studies by Nguyen *et al.* (54) have shown that *Mag* can maintain the stability of microtubules in axons in the peripheral nervous system and serves as a marker of axonal resistance to degeneration. Nerve injury reduced *Mag* protein levels, whereas *Trpa1* inhibitor treatment increased *Mag* levels, suggesting *Trpa1* pathway regulation of *Mag* expression. On the one hand, *Trpa1* inhibition reduces neuroinflammation and pain sensitivity, enhancing *Mag* stability and expression. *Trpa1* likely directly influences *Mag* synthesis in Schwann cells. Its activation engages multiple signaling pathways, which may potentially regulate *Mag* expression. In short, the inhibition of the *Trpa1* pathway might enhance axonal stability and resistance to degeneration by mitigating the adverse effects of the injury environment on axons. Our proposed conceptualization of the role of *Trpa1* in mediating neuropathic pain is depicted in Figure 7.

## Conclusion

Our study shows that the *Trpa1* pathway in neurons and dedifferentiated Schwann cells regulates the synthesis and release of *Cxcl1*, recruiting *Cxcr2*-positive macrophages to the site of nerve injury, thus mediating neuropathic pain. In addition, *Trpa1* mediates reduction of *Mag*, weakening axonal microtubule stability and reducing resistance to degeneration. These findings offer new therapeutic strategies and theoretical insights for treating neuropathic pain.

## Experimental procedures

### Animals

The experiment utilized healthy adult male C57BL/6J mice, aged 8 weeks, weighing 20 to 25 g, purchased from the Guangdong Medical Laboratory Animal Center. All animal studies/procedures have been approved by the Institutional Animal Care and Use Committee of The Second Affiliated Hospital of Guangzhou Medical University and performed in accordance with the ethical standards. The acceptance number is A2021-028. All mice were housed at the Experimental Animal Center affiliated with the Second Affiliated Hospital of Guangzhou Medical University. They were randomly assigned to groups and maintained under controlled environmental conditions with a temperature of 21 ± 1 °C, a 12-h light–dark cycle, and humidity maintained at 50% to 60%. Food and water were provided ad libitum.

### SNI model

All instruments were sterilized. First, mice were induced anesthesia using 2 to 3% isoflurane until fully anesthetized and then transferred to a 37 °C heated surgical board under this anesthesia. The surgical area was shaved from the buttock toward thigh and knee by electric shaver. The animal was positioned on its right side on the operating table, and the left hind limb was placed on a small platform, securing the leg with adhesive tape. The surgical site was sterilized with iodine solution. Using the left thumb to locate the knee, a 1 cm longitudinal incision was made near the proximal end of the thigh with a surgical blade. Blunt dissection of the muscle layer reveals the sciatic nerve directly. Carefully separating the muscles, the area of the sciatic nerve branches was identified, with the tibial nerve being the smallest branch of the three branches, branching to the right on the left leg. The other two branches (peroneal nerve and sural nerve) were sutured around with 6-0 sutures, taking extreme care not to touch the peroneal branch to avoid any damage and keep the branch intact. The nerve below the suture was grasped with forceps, and a small pair of scissors was used to transect the nerve just below the knot. Approximately 1 mm of the distal end of the nerve was cut, and the end of the suture was cut off with fine scissors, taking care not to pull the proximal nerve during the operation. The muscle layer and skin were gently closed and disinfected to keep the wound clean. In the Sham surgery group, muscles and skin were sutured after exposing the sciatic nerve, with all other procedures identical to those in the experimental group. Postoperatively, the animals were placed in a constant temperature box to maintain body temperature until they regained consciousness, ensuring easy access to water and food for the mice.

### RNA sequencing and analysis

The process involves RNA extraction and detection, library construction and quality control, sequencing, and bioinformatics analysis. For RNA extraction, nanodrop was used for concentration and purity measurement, whereas Agilent Bioanalyzer and RNA gel electrophoresis were employed for integrity verification. Library construction starts with mRNA isolation from ≥1 μg total RNA, followed by complementary DNA (cDNA) synthesis, end repair, and adapter ligation. Quality control was conducted using Agilent Bioanalyzer and PicoGreen quantification, with PCR amplification and size selection using AMPure XP beads. Sequencing was performed on an Illumina platform in PE150 mode. Bioinformatics analysis involves quality check, data filtering, alignment using HISAT2, read count estimation, normalization using fragments per kilobase of transcript per million mapped fragments, differential expression analysis with DEGSeq, GO enrichment analysis with topGO, and KEGG pathway enrichment analysis with clusterProfiler.

### Behavioral evaluation of pain

Mechanical pain withdrawal threshold was measured using the von Frey filament test. Prior to the initial test, mice were acclimatized to the testing environment for three consecutive days, with three sessions each day lasting 60 min. Before each behavioral test, mice were allowed to acclimate on the testing platform for 30 min until they were calmed down. All behavioral tests in this study focused on the left hind paw of the mice. To assess mechanical pain withdrawal threshold, mice were placed in a transparent rectangular acrylic box (10 ∗ 10 ∗ 15 cm on a 1 ∗ 1 cm wire mesh for 30 min to acclimate. Once the mice were calmed down, von Frey filaments ranging from 0.04 g to 2.0 g were vertically applied to the lateral one-third of the mouse's hind paw. The filament was bent into a "S" shape and maintained for 5 s. If the mouse exhibited paw withdrawal, licking, or jumping behavior, it was considered a positive response. Each round of testing started with the 0.04 g filament, repeated five times. There was at least a 15-s interval between each stimulation. If the positive response occurred three or more times within five repetitions, that filament strength was recorded as the mechanical pain withdrawal threshold for that round. Otherwise, the filament strength was increased by one unit for the next round of testing, up to a maximum of 2.0 g. If the positive response to the 2.0 g filament was still less than three times, it was recorded as 2.0 g, and no further increase in filament strength was performed to avoid injury to the mouse's paw. This process was repeated for five rounds of testing, and the average value of the five rounds was recorded as the mechanical pain withdrawal threshold for that session.

### Immunofluorescence

Following deep anesthesia with isoflurane, the left sciatic nerve was harvested after cardiac perfusion with precooled PBS solution and fixation with 4% paraformaldehyde. Different dehydration and embedding methods were employed for frozen and paraffin sections. Frozen sections underwent dehydration in 30% sucrose solution followed by embedding in optimal cutting temperature compound (7 μm), whereas paraffin sections were dehydrated in ethanol and subsequently embedded in paraffin wax (3 μm). Frozen sections were washed with 0.01 M PBS solution, treated with 0.3% Triton X-100 solution for membrane permeabilization for 10 min, washed again with 0.01 M PBS solution, and then incubated in 3% bovine serum albumin solution for 1 h for blocking. Subsequently, primary antibodies (antibodies used are detailed in Table 1) were applied and incubated overnight at 4 °C, followed by incubation with fluorescent secondary antibodies for 1 h under light-shielded conditions. Finally, sections were mounted with 4′,6-diamidino-2-phenylindole–containing mounting medium and observed under a fluorescence microscope. For paraffin sections, deparaffinization and antigen retrieval were performed using gradient ethanol and antigen retrieval solution before membrane permeabilization.

**Table 1:** List of antibodies used for Western blotting and immunofluorescence

Antibody	Source and catalog no.	WB	IF
TRPA1 antibody	Affinity, DF 13269	1:1000	—
α-Tubulin antibody	Proteintech, 66031-1-Ig	1:20,000	—
TRPA1 antibody	Novus Biologicals, NB110-40763	—	1:1000
GFAP antibody	Cell Signaling Technology, 12389	1:1000	—
CXCL1 antibody	Thermo Fisher Scientific, PA5-86508	1:1000	1:100
GAPDH antibody	Proteintech, 60004-1-Ig	1:50,000	—
GFAP antibody	Proteintech, 60190-1-Ig	—	1:500
NF-H/NF200 antibody	Proteintech, 60331-1-Ig	—	1:200
CXCR2 antibody	Proteintech, 20634-1-AP	—	1:100
IBA1 antibody	Abcam, ab178847	—	1:1000
MAG antibody	Abcam, ab277524	1:1000	—
β-actin antibody	Affinity, T0022	1:10,000	—
MAG antibody	Proteintech, 66709-1-Ig	—	1:500
Goat anti-Mouse IgG 488	Thermo Fisher Scientific, A-11001	—	1:2000
Goat anti-Rabbit IgG 594	Thermo Fisher Scientific, A-11037	—	1:500
Goat anti-Mouse IgG H&L-HRP	Abcam, ab205719	1:10,000	—
Goat anti-Rabbit IgG H&L-HRP	Abcam, ab205718	1:10,000	—

### RT–qPCR

First, tissue grinding was performed by placing the collected sciatic nerve tissue with grinding beads in TRIzol on ice and using a precooled tissue grinder set to a frequency of 60 Hz, with a 10-s pause every 30 s, for a total of 10 min to ensure thorough grinding. Next is total RNA extraction, where the ground tissue solution was added to TRIzol and left to stand at 4 °C in a refrigerator. After centrifugation, the supernatant containing RNA was collected, and RNA precipitation was achieved by adding isopropanol followed by centrifugation. The RNA pellet obtained was then washed with ethanol, dried, and dissolved to obtain total RNA. Subsequently, cDNA synthesis was conducted according to the protocol of HiScript III RT SuperMix, wherein total RNA was reverse transcribed into cDNA and stored at −80 °C. Finally, target gene expression determination was carried out using ChamQ SYBR qPCR Master Mix for real-time fluorescence qPCR. Using GAPDH as the internal reference gene, cDNA was synthesized, and PCR amplification was performed to determine the Ct value for calculating the relative expression level of the target gene. All steps were performed on ice or at 4 °C to maintain RNA integrity. Gene primers are listed in Table 2.

**Table 2:** List of primers used for RT–qPCR

Gene	Forward primer: 5′-3′	Reverse primer: 5′-3′
GAPDH	*AGGTCGGTGTGAACGGATTTG*	TGTAGACCATGTAGTTGAGGTCA
CCL7	CCTGGGAAGCTGTTATCTTCAAG	CCTCCTCGACCCACTTCTGA
DOK2	GGTTCGCAGCCGTGTTATATG	CCCGTAAGCAGTCACTGAGG
TLR5	GCAGGATCATGGCATGTCAAC	ATCTGGGTGAGGTTACAGCCT
CXCL1	ACTGCACCCAAACCGAAGTC	TGGGGACACCTTTTAGCATCTT
GFAP	GGGGCAAAAGCACCAAAGAAG	GGGACAACTTGTATTGTGAGCC
TRIM6	ACCCCTGAGCATTGATTGTGG	CAGGTGCCGATTAGGACGG

### Western blotting

The sciatic nerve was dissected from the mouse, and protein extraction was carried out using radioimmunoprecipitation assay buffer supplemented with protease inhibitors, phosphatase inhibitors, and PMSF. Protein concentration was determined using the BCA Protein Assay Kit (ThermoFisher), and based on the molecular weight of the target protein, the appropriate concentration of SDS-PAGE gel was selected. Subsequently, electrophoresis and transfer of samples from the SDS-PAGE gel to a polyvinylidene difluoride membrane were performed using an electrophoresis apparatus. The membrane was then blocked with 5% milk buffer at room temperature for 1 h, followed by overnight incubation with primary antibody (antibodies used are detailed in Table 1) at 4 °C. After washing the membrane with Tris-buffered saline with Tween-20, it was then incubated with a secondary antibody conjugated with horseradish peroxidase at room temperature for 1 h. Chemiluminescent detection was carried out using an ECL substrate (ThermoFisher) after incubation.

### Statistical analysis

All experimental results in this study were presented as mean ± SD. Graphs were generated using GraphPad Prism 8 (GraphPad Software) and Adobe Illustrator (Adobe Systems), whereas statistical analyses were performed using ImageJ and GraphPad Prism 8 software. Group differences were compared using *t* tests or one-way ANOVA; when *p* < 0.05, the differences between groups were considered statistically significant.

## Data availability

Data will be made available on request.

## Conflict of interest

The authors declare that they have no conflicts of interest with the contents of this article.
